# The association between dietary index for gut microbiota and colorectal cancer prevalence in US adults: Findings from NHANES 2003 to 2023

**DOI:** 10.1097/MD.0000000000049915

**Published:** 2026-07-31

**Authors:** Xingyong Feng, Jiaojiao Long, Wei Wang

**Affiliations:** a Department of Oncology, Huanggang Central Hospital, Huanggang, Hubei, China; b School of Medicine, Wuhan University of Science and Technology, Wuhan, China; c Department of Gastroenterology, Huanggang Central Hospital, Huanggang, Hubei, China.

**Keywords:** colorectal cancer, DI-GM, dietary index, gut microbiota, NHANES

## Abstract

Emerging evidence suggests that diet plays a crucial role in shaping gut microbiota, which may influence the risk of colorectal cancer (CRC). This study examined the association between the dietary index for gut microbiota (DI-GM) and CRC prevalence in a nationally representative sample of US adults. This cross-sectional analysis used data from 31,547 adults aged ≥20 years participating in the National Health and Nutrition Examination Survey 2003 to 2023. Weighted logistic regression models estimated odds ratios and 95% confidence intervals for CRC across DI-GM scores, adjusting for demographic, lifestyle, and clinical factors. The mean age of participants was 46.2 ± 17.1 years, and 316 individuals self-reported a CRC diagnosis. CRC cases had significantly lower DI-GM scores than non-cases (3.7 ± 1.5 vs 4.3 ± 1.7, *P* < .001). Higher DI-GM scores were inversely associated with CRC prevalence (adjusted odds ratio = 0.89, 95% confidence interval = 0.86–0.98). When analyzed categorically, participants with DI-GM scores ≥6 had 30% lower odds of CRC than those with scores ≤3. Diets that promote a healthier gut microbiota composition are associated with a lower prevalence of CRC.

## 1. Introduction

Colorectal cancer (CRC) remains one of the most prevalent and lethal malignancies worldwide, ranking as the third most commonly diagnosed cancer and the second leading cause of cancer-related mortality globally.^[1]^ In the United States, CRC continues to pose a significant public health challenge, with both incidence and mortality rates remaining substantial despite advances in screening and treatment.^[2]^ Mounting evidence suggests that environmental and lifestyle factors, particularly diet, play a critical role in CRC etiology and progression.^[3]^

Dietary patterns have long been recognized as major determinants of CRC risk, with high intake of red and processed meats, saturated fats, and ultra-processed foods consistently associated with increased CRC incidence, while diets rich in fiber, fruits, vegetables, and whole grains are linked to reduced risk.^[4]^ The biological mechanisms underlying these associations are increasingly attributed to the gut microbiota, a complex and dynamic community of microorganisms inhabiting the human gastrointestinal tract. The gut microbiome not only modulates host metabolism, immune function, and epithelial integrity but also mediates the production of bioactive metabolites, such as short-chain fatty acids (SCFAs), secondary bile acids, and other microbial byproducts that can influence colorectal carcinogenesis.^[5]^

Recent research has demonstrated that diet is a primary modifiable factor shaping the composition and function of the gut microbiota. For example, high-fiber diets promote the proliferation of beneficial bacteria capable of fermenting complex carbohydrates into SCFAs, such as butyrate, which exerts anti-inflammatory and antineoplastic effects on colonic epithelial cells.^[6]^ Conversely, Western dietary patterns characterized by low fiber and high fat intake can foster dysbiosis, increase the abundance of pro-carcinogenic bacteria (e.g., *Fusobacterium nucleatum*, *Bacteroides fragilis*), and elevate the production of genotoxic metabolites, thereby enhancing CRC risk.^[7]^ Animal and human studies have further highlighted the rapid responsiveness of the gut microbiome to dietary interventions, with shifts in microbial diversity and metabolic output observed following short- and long-term changes in dietary intake.^[8]^

Epidemiological and experimental studies have consistently reported that alterations in the gut microbiota are associated with CRC development and progression.^[9,10]^ Meta-analyses of metagenomic datasets have identified CRC-associated microbial signatures, including increased abundance of *Fusobacterium nucleatum*, *Peptostreptococcus stomatis*, and *Gemella morbillorum*, alongside functional changes in microbial metabolic pathways.^[9,11]^ Furthermore, dietary indices specifically designed to reflect gut microbiota-friendly eating patterns have emerged as promising tools for assessing the diet-microbiome-CRC axis in population studies. However, most existing research has been limited by cross-sectional designs, small sample sizes, or a lack of comprehensive dietary and microbiome data over time.^[12,13]^

Given the critical interplay between diet, gut microbiota, and CRC risk, large-scale, population-based studies are needed to elucidate these relationships in diverse cohorts. The National Health and Nutrition Examination Survey (NHANES) offers a unique opportunity to investigate these associations in a representative sample of US adults, with extensive dietary, health, and demographic data collected over 2 decades. This study aims to examine the association between a dietary index for gut microbiota and CRC risk among US adults using NHANES 2003 to 2023 data, thereby advancing our understanding of modifiable dietary strategies for CRC prevention in the general population.

## 2. Materials and methods

### 2.1. Study population

This study used data from the NHANES, a continuous, cross-sectional survey conducted in the United States to assess the health and nutritional status of the noninstitutionalized civilian population. NHANES employs a complex, multistage probability sampling design and is administered by the National Center for Health Statistics. All participants provided written informed consent, and the study protocol was approved by the relevant institutional review board. For this analysis, data from NHANES cycles spanning 2003 to 2023 were included. Of the 98,551 individuals initially evaluated, participants were excluded if they were under 20 years of age, reported a history of any malignancy other than CRC, or had missing data for key covariates including dietary intake, sociodemographic variables, and clinical characteristics. After applying these exclusion criteria, the final analytic sample comprised 31,547 adults. CRC status was determined based on self-reported physician diagnosis. Participants were asked: “Have you ever been told by a doctor or other health professional that you had cancer or a malignancy of any kind?” “What type of cancer was it?” Only participants who identified colon and rectal cancer were included in the study. CRC status was based on self-reported physician diagnosis. Self-reported cancer outcomes in NHANES have been widely used in epidemiological studies and generally demonstrate moderate to high validity for major cancer types.^[14,15]^

### 2.2. Dietary index for gut microbiota

In the NHANES study, dietary intake data were collected through two 24-hour dietary recall interviews. The first recall was conducted in person at the Mobile Examination Center, followed by a second recall via telephone approximately 3 to 10 days later. The dietary index for gut microbiota (DI-GM) was calculated using data from NHANES by averaging the results of the 2 dietary recall interviews, thereby minimizing potential inconsistencies related to recall frequency. The DI-GM reflects a composite dietary pattern based on the intake of 14 food components identified in the literature as beneficial or detrimental to gut microbiota health. Beneficial components included avocado, broccoli, chickpeas, coffee, cranberries, fermented dairy products, dietary fiber, green tea, soy, and whole grains. Detrimental components included red meat, processed meat, refined grains, and high-fat diets (≥40% of total energy intake from fat). For beneficial components, a score of 1 was assigned if intake was equal to or above the sex-specific median, and 0 if below. For detrimental components, a score of 1 was assigned for intake below the median and 0 for intake at or above the median. Since the NHANES 24-hour recall data do not include specific tea consumption, DI-GM scores range from 0 to 13, with higher scores reflecting a dietary pattern more favorable to gut microbiota.^[16]^ More detailed information about the composition and calculation of DI-GM can be found in Table S1, Supplemental Digital Content 1. DI-GM was examined both as a continuous variable and as a categorical variable (0–3, 4, 5, and ≥6) in the analyses.

### 2.3. Covariates

Covariates were selected based on prior evidence and included sociodemographic, socioeconomic, and health behavioral characteristics. Sociodemographic factors comprised age (continuous), gender (male or female), and race/ethnicity (non-Hispanic White, non-Hispanic Black, Hispanic, and other). Socioeconomic indicators included marital status (married/living with partner vs not married), poverty-income ratio (PIR; categorized as low income <1.30, middle income 1.30–3.49, and high income ≥3.50), and educational attainment (less than high school, high school graduate, and college or above).

Health behavior variables included smoking status (smoker vs nonsmoker), diabetes status (yes or no), physical activity level (inactive, moderately active, and highly active), alcohol consumption (nondrinker, moderate drinker, and heavy drinker), and total energy intake (kcal/d, continuous). Body mass index (BMI) was calculated from measured height and weight and treated as a continuous variable in all models. These covariates were included in multivariable models to control for potential confounding in the association between DI-GM and CRC.

### 2.4. Statistical analysis

Descriptive statistics were used to summarize baseline characteristics of participants by CRC status. Continuous variables were presented as means with standard deviations and compared using independent sample *t* tests after assessing normality and homogeneity of variances. Categorical variables were expressed as frequencies (percentages) and compared using the chi-square test. Multivariable logistic regression models were used to estimate odds ratios (ORs) and 95% confidence intervals (CIs) for the association between DI-GM and CRC. Model 1 was unadjusted. Model 2 adjusted for age, gender, race/ethnicity, PIR, BMI, diabetes status, smoking status, education level, physical activity, alcohol consumption, and total energy intake. Trend tests were conducted by modeling the median value of each DI-GM category as a continuous variable. All regression analyses accounted for the complex NHANES sampling design using appropriate survey weights (WTMEC2YR), strata (SDMVSTRA), and primary sampling units (SDMVPSU). Combined survey weights were constructed according to NHANES analytic guidelines for multiple cycles (2003–2023) by dividing the 2-year weights by the number of cycles included. Subgroup analyses were performed stratified by age, gender, race/ethnicity, diabetes status, smoking status, and BMI categories to assess the consistency of associations across different population segments. Missing data on covariates were handled using multiple imputation with chained equations under the assumption of missing at random. A total of 5 imputed datasets were generated, and estimates were combined using Rubin’s rules. The imputation model included all variables used in the main analyses, including exposure, outcome, and covariates. Interaction terms were tested in the multivariable models to evaluate statistical interactions (*P* for interaction < .10 was considered suggestive). All statistical analyses were performed using R version 4.3.3 and SPSS version 21.0 (IBM Corp.), with a two-sided *P*-value < .05 considered statistically significant.

## 3. Results

### 3.1. Participant characteristics

A total of 31,547 adults were included, of whom 316 had a history of CRC. Table 1 shows participant characteristics. Individuals with CRC were significantly older than those without CRC (62.3 ± 12.4 vs 45.9 ± 17.0 years, *P* < .001). CRC prevalence was higher among participants with lower income (PIR < 1.30), non-Hispanic Black or other ethnicity, unmarried status, smoking history, diabetes, lower education, physical inactivity, and lower alcohol consumption (all *P* < .05). Mean BMI was slightly higher among CRC cases compared to non-cases (29.6 ± 6.8 vs 28.7 ± 6.2 kg/m^2^, *P* = .017).

**Table 1:** Characteristics of the study participants, NHANES (2003–2023).

Characteristic	Overall (N = 31,547)	Non-CRC (N = 31,231)	CRC (N = 316)	*P* value
Age (yr)	46.2 ± 17.1	45.9 ± 17.0	62.3 ± 12.4	<.001
Gender				
Male	15,812 (50.1%)	15,651 (50.1%)	161 (50.9%)	.689
Female	15,735 (49.9%)	15,580 (49.9%)	155 (49.1%)	
PIR (%)				
<1.30	7245 (23.0%)	7138 (22.9%)	107 (33.9%)	.041
1.30–3.50	14,738 (46.7%)	14,602 (46.8%)	136 (43.0%)	
≥3.50	9564 (30.3%)	9491 (30.4%)	73 (23.1%)	
Education (%)				
Less than high school	6812 (21.6%)	6690 (21.4%)	122 (38.6%)	<.001
High school graduate	7984 (25.3%)	7905 (25.3%)	79 (25.0%)	
College or above	16,751 (53.1%)	16,636 (53.3%)	115 (36.4%)	
Physical activity (%)				
Inactive	9215 (29.2%)	9036 (28.9%)	179 (56.6%)	<.001
Moderately active	11,487 (36.4%)	11,384 (36.4%)	103 (32.6%)	
Highly active	10,845 (34.4%)	10,811 (34.7%)	34 (10.8%)	
Ethnicity (%)				
Hispanic	5672 (18.0%)	5623 (18.0%)	49 (15.5%)	.021
Non-Hispanic White	16,048 (50.9%)	15,914 (50.9%)	134 (42.4%)	
Non-Hispanic Black	6125 (19.4%)	6048 (19.4%)	77 (24.4%)	
Other	3702 (11.7%)	3646 (11.7%)	56 (17.7%)	
Marital (%)				
Married/living with partner	17,542 (55.6%)	17,392 (55.7%)	150 (47.5%)	.038
Other	14,005 (44.4%)	13,839 (44.3%)	166 (52.5%)	
Smoking status (%)				
Smoker	13,094 (41.5%)	12,884 (41.3%)	210 (66.5%)	<.001
Nonsmoker	18,453 (58.5%)	18,347 (58.7%)	106 (33.5%)	
Diabetes (%)				
Yes	4258 (13.5%)	4132 (13.2%)	126 (39.9%)	<.001
No	27,289 (86.5%)	27,099 (86.8%)	190 (60.1%)	
Alcohol consumption (%)				
Nondrinker	9462 (30.0%)	9294 (29.8%)	168 (53.2%)	.002
Moderate drinker	14,926 (47.3%)	14,813 (47.4%)	113 (35.8%)	
Heavy drinker	7159 (22.7%)	7124 (22.8%)	35 (11.0%)	
Total energy intake (kcal/d)	2065 ± 742	2073 ± 738	1932 ± 695	.03
Body mass index (kg/m^2^)	28.8 ± 6.3	28.7 ± 6.2	29.6 ± 6.8	.017
DI-GM, mean ± SD	4.2 ± 1.8	4.3 ± 1.7	3.7 ± 1.5	<.001
Beneficial to gut microbiota, mean ± SD	2.6 ± 1.2	2.7 ± 1.1	1.9 ± 1.0	<.001
Unbeneficial to gut microbiota, mean ± SD	1.5 ± 0.9	1.5 ± 0.8	1.7 ± 0.7	.12

All continuous variables are presented as means (SDs) and categorical variables are presented as numbers (%). *P* values were calculated based on independent sample *t* test or chi-square test.

CRC = colorectal cancer, DI-GM = dietary index for gut microbiota, NHANES = National Health and Nutrition Examination Survey, PIR = poverty-income ratio, SD = standard deviation.

The overall mean DI-GM score was 4.2 ± 1.8, with significantly lower values in CRC participants (3.7 ± 1.5) than in non-CRC participants (4.3 ± 1.7, *P* < .001). CRC cases also reported lower intake of foods beneficial to gut microbiota (1.9 ± 1.0 vs 2.7 ± 1.1, *P* < .001), while unbeneficial food intake was marginally higher among CRC cases (1.7 ± 0.7 vs 1.5 ± 0.8, *P* = .12).

### 3.2. Association between DI-GM and CRC

Logistic regression results are presented in Table 2. In unadjusted analyses (model 1), each unit increase in DI-GM was associated with a 14% lower odds of CRC (OR = 0.86, 95% CI = 0.81–0.94, *P* < .001). After adjusting for age, gender, race/ethnicity, PIR, BMI, diabetes, smoking, education, physical activity, alcohol intake, and total energy intake (model 2), the inverse association remained significant (OR = 0.92, 95% CI = 0.88–0.99, *P* = .021). Compared to individuals with the lowest DI-GM scores (0–3), those in the highest category (≥6) had lower odds of CRC in both unadjusted (OR = 0.66, 95% CI = 0.50–0.88) and adjusted models (OR = 0.74, 95% CI = 0.54–0.96). Higher consumption of beneficial foods was also associated with reduced CRC odds in unadjusted (OR = 0.83, 95% CI = 0.71–0.95, *P* = .008) and adjusted models (OR = 0.89, 95% CI = 0.76–0.99, *P* = .042), while intake of unbeneficial foods showed no significant association.

**Table 2:** Association between DI-GM and colorectal cancer, NHANES 2003 to 2023.

Exposures	Model 1*		Model 2**	
	OR (95% CI)	*P* value	OR (95% CI)	*P* value
DI-GM	0.86 (0.81–0.94)	<.001	0.92 (0.88–0.99)	.021
0–3	1	–	1	–
4	0.88 (0.68–1.13)	.306	0.94 (0.71–1.25)	.676
5	0.77 (0.59–1.01)	.061	0.85 (0.64–1.10)	.198
≥6	0.66 (0.50–0.88)	.004	0.74 (0.54–0.96)	.037
*P* for trend		<.001		.018
Beneficial to gut microbiota	0.83 (0.71–0.95)	.008	0.89 (0.76–0.99)	.042
Unbeneficial to gut microbiota	1.09 (0.91–1.30)	.336	1.03 (0.85–1.25)	.721

CI = confidence interval, DI-GM = dietary index for gut microbiota, NHANES = National Health and Nutrition Examination Survey, OR = odds ratio, PIR = poverty-income ratio.

* Model 1 not adjusted.

** Model 2 adjusted for age, gender, ethnicity, PIR, diabetes, body mass index, smoking, education level, physical activity, alcohol consumption, and total energy intake.

### 3.3. Subgroup and sensitivity analyses

Subgroup analyses (Table 3) revealed generally consistent inverse associations between DI-GM and CRC across age, ethnicity, smoking, diabetes, and BMI subgroups. Although the protective association appeared stronger among adults ≥60 years (OR = 0.71, 95% CI = 0.53–0.96) and non-Hispanic Whites (OR = 0.72, 95% CI = 0.54–0.96), tests for interaction were not statistically significant. Similarly, the inverse associations were evident among both smokers (OR = 0.74, 95% CI = 0.55–0.99) and nonsmokers (OR = 0.68, 95% CI = 0.51–0.91), and across BMI categories, with the strongest effect observed in participants with BMI 25 to 29.9 kg/m^2^ (OR = 0.69, 95% CI = 0.50–0.95).

**Table 3:** Associations between DI-GM and colorectal cancer, stratified by selected factors, NHANES 2003 to 2023.

Characteristic	OR (95% CI)	*P* value	*P* for interaction
Age			
<40	0.88 (0.62–1.26)	.488	.118
40–59	0.75 (0.56–1.01)	.058	
≥60	0.71 (0.53–0.96)	.027	
Gender			
Male	0.94 (0.82–1.1)	.083	.329
Female	0.91 (0.85–1)	.052	
Ethnicity			
Hispanic	0.84 (0.59–1.21)	.358	.21
Non-Hispanic White	0.72 (0.54–0.96)	.027	
Non-Hispanic Black	0.91 (0.66–1.27)	.590	
Other	0.79 (0.53–1.19)	.255	
Smoking status			
Smoker	0.74 (0.55–0.99)	.045	.149
Nonsmoker	0.68 (0.51–0.91)	.010	
Diabetes status			
Yes	0.97 (0.93–1.05)	.076	.33
No	0.95 (0.91–1.02)	.062	
Body mass index			
<24.9	0.72 (0.49–1.06)	.094	.205
25–29.9	0.69 (0.50–0.95)	.023	
≥30	0.76 (0.57–1.02)	.069	

Each stratification was adjusted for age + sex + ethnicity + diabetes + BMI + smoking status. The strata variable was not included when stratifying by itself.

CI = confidence interval, DI-GM = dietary index for gut microbiota, NHANES = National Health and Nutrition Examination Survey, OR = odds ratio.

To handle missing data, we applied multiple imputation by chained equations, generating 5 imputed datasets. Multivariate logistic regression analyses on these imputed datasets showed associations between DI-GM and CRC that were consistent with the primary complete-case analysis. The effect sizes and directions remained similar, reinforcing the robustness of our findings. Detailed results of the multiple imputation analyses are provided in Table S2, Supplemental Digital Content 2.

## 4. Discussion

In this large, nationally representative sample of 31,547 US adults, including 316 individuals with a history of CRC, we observed a significant inverse association between adherence to the DI-GM and CRC prevalence. Specifically, each one-unit increase in DI-GM score was associated with an 8% reduction in the prevalence of CRC after adjusting for key confounders. Participants in the highest DI-GM category (score ≥ 6) had approximately 26% lower prevalence of CRC compared to those in the lowest category (0–3). These associations remained consistent across multiple subgroups, including age, ethnicity, smoking status, diabetes, and BMI, highlighting the broad relevance of gut microbiota-targeted dietary patterns in CRC prevention.

Mechanistically, the protective effect of higher DI-GM scores likely reflects multiple interrelated biological pathways. First, diets scoring high on the DI-GM are rich in fiber, polyphenols, and other microbiota-accessible carbohydrates that promote the growth of beneficial saccharolytic bacteria such as *Faecalibacterium prausnitzii* and *Roseburia* spp. These bacteria produce SCFAs, particularly butyrate, which serves as a key epigenetic regulator by inhibiting histone deacetylases and modulating gene expression involved in cell proliferation and apoptosis.^[17,18]^ Butyrate also exerts potent anti-inflammatory effects by enhancing regulatory T-cell differentiation and suppressing pro-inflammatory Th17 responses, which aligns with our findings of reduced systemic inflammation markers among individuals with higher DI-GM scores.^[19,20]^ These immunomodulatory effects contribute to maintaining colonic epithelial homeostasis and preventing malignant transformation.^[5,17]^

Second, the DI-GM emphasizes consumption of foods that support intestinal barrier integrity. The marginally higher intake of unbeneficial foods observed among CRC cases may contribute to disruption of tight junction proteins, increasing gut permeability and facilitating translocation of endotoxins that activate pro-inflammatory pathways such as TLR4/MyD88 and NF-κB signaling.^[19,21]^ In contrast, polyphenol-rich foods included in the DI-GM promote the growth of mucin-degrading bacteria like *Akkermansia muciniphila*, which enhance mucosal barrier function and reduce endotoxin-induced inflammation.^[22]^ This mechanism is particularly relevant for subpopulations with metabolic comorbidities such as diabetes, who are known to have compromised gut barrier function.^[5,23]^

Third, the DI-GM may mitigate exposure to dietary carcinogens and their microbial metabolites. For example, cruciferous and allium vegetables, key components of the DI-GM, contain bioactive compounds that induce detoxifying enzymes and inhibit the growth of pro-carcinogenic bacteria such as *Fusobacterium nucleatum*, which has been implicated in CRC tumorigenesis through activation of β-catenin signaling and modulation of the tumor immune microenvironment.^[24–26]^ The observed stronger protective association among smokers may reflect the ability of DI-GM-aligned diets to counteract tobacco-related carcinogenic pathways via enhanced detoxification and microbiota modulation.^[22]^

Our subgroup analyses revealed that the protective association of DI-GM with CRC was consistent across age groups, with a somewhat stronger effect in adults aged 60 years and older. This finding is consistent with the notion that age-related declines in microbial diversity and butyrate production increase CRC susceptibility, which can be partially reversed by adherence to microbiota-friendly diets.^[5,27]^ Similarly, the association was robust across ethnic groups, although the slightly attenuated effect among non-Hispanic Black participants may reflect disparities in access to healthy foods and differences in microbiome composition driven by socioeconomic factors.^[5]^ Subgroup and interaction analyses were conducted to explore potential effect modification. Given the number of comparisons and the use of a conventional significance threshold without formal adjustment for multiple testing, these analyses should be considered exploratory. Therefore, subgroup-specific findings should be interpreted with caution.

Compared to previous studies, our findings reinforce and extend the evidence linking diet, gut microbiota, and CRC prevalence. Unlike traditional dietary indices such as the Healthy Eating Index, the DI-GM specifically targets foods known to modulate gut microbial ecology, which may explain its stronger association with CRC risk reduction.^[5,26]^ Our results also complement intervention studies demonstrating that high-fiber, polyphenol-rich diets reduce colorectal adenoma recurrence and favorably alter gut microbial profiles.^[28]^

Despite the strengths of our study, including a large, diverse sample and comprehensive adjustment for confounders, some limitations should be acknowledged. The cross-sectional design of NHANES limits causal inference. Both dietary intake and CRC diagnosis were assessed via self-report, which may introduce measurement error. For dietary intake, this may bias effect estimates, while for CRC, misclassification is likely non-differential with respect to dietary exposure, potentially biasing associations toward the null. However, differential misclassification related to healthcare access or socioeconomic status cannot be excluded. Moreover, we did not have direct microbiome sequencing data to confirm microbial shifts associated with DI-GM adherence. Nevertheless, the biological plausibility of our findings is supported by extensive mechanistic literature and consistency with metabolomic studies linking high DI-GM diets to reduced pro-carcinogenic metabolites.^[17,22]^ Given the cross-sectional design and the use of a prevalent outcome (“ever diagnosed with CRC”), causal inference remains unattainable. Reverse causation is a major concern, as individuals diagnosed with CRC may have altered their dietary habits post-diagnosis. Furthermore, survivorship bias may be present, since prevalent cases reflect only those who survived long enough to participate in NHANES and may systematically differ from incident cases. Consequently, the observed associations should be interpreted as reflecting CRC prevalence rather than incidence or risk.

In terms of clinical and public health implications, our findings suggest that promoting dietary patterns aligned with the DI-GM could be an effective strategy for CRC prevention across diverse populations. Personalized nutrition interventions incorporating microbiome profiling may further optimize preventive efforts, particularly in high-risk groups such as older adults, smokers, and individuals with metabolic disorders.^[5,23]^ Public health policies aimed at increasing accessibility and affordability of microbiota-friendly foods, including fiber-rich fruits, vegetables, and whole grains, are warranted to reduce CRC burden at the population level.^[28,29]^

Future research should focus on prospective cohort studies with serial microbiome and metabolome assessments to elucidate causal pathways and temporal dynamics.^[30]^ Randomized controlled trials testing DI-GM-based dietary interventions with CRC-related biomarkers as endpoints are also needed to establish efficacy. Integration of multi-omics approaches and machine learning may enable precision nutrition strategies tailored to individual microbiome profiles and genetic backgrounds.^[30]^

## 5. Conclusion

Our study provides evidence of an inverse association between adherence to a gut microbiota-targeted dietary index and the prevalence of CRC in US adults. Therefore, given the cross-sectional nature of this study, our findings are hypothesis-generating and future longitudinal studies are recommended to confirm these findings and to better assess potential causal relationships.

## Acknowledgments

We would like to thank all NHANES staff and participants.

## Author contributions

**Formal analysis:** Xingyong Feng.

**Validation:** Xingyong Feng.

**Investigation:** Jiaojiao Long.

**Methodology:** Jiaojiao Long.

**Software:** Jiaojiao Long, Wei Wang.

**Data curation:** Wei Wang.

**Writing – original draft:** Xingyong Feng, Jiaojiao Long, Wei Wang.

**Writing – review & editing:** Xingyong Feng, Jiaojiao Long, Wei Wang.




