# Supplementary material for: The association between dietary index for gut microbiota and colorectal cancer prevalence in US adults: Findings from NHANES 2003 to 2023
Source: Medicine (Baltimore). 2026 Jul 31;105(31):e49915. doi: 10.1097/MD.0000000000049915 (PMC13433049; doi:10.1097/MD.0000000000049915)
Supplement: Supplementary file 1 [file medi-105-e49915-s001.docx]

| **Table S1. Components of the DI-GM.** | | |
| --- | --- | --- |
| **Component** | **Included Foods within the Component** | **Scoring** |
| **Beneficial to gut microbiota** |  |  |
| Avocados | Avocados | For each component, a score of 1 if consumption at or above the sex-specific median, else 0 |
| Broccoli | Broccoli |  |
| Chickpea | Chickpeas |  |
| Coffee | Coffee |  |
| Cranberries | Cranberries |  |
| Fermented dairy | Yogurt, cheese, kefir, sour cream, buttermilk |  |
| Fiber | Not applicable |  |
| Soybean | Soy products—Soy milk, Tofu |  |
| Whole grains | Grains defined as whole grains, containing the entire grain kernel―the bran, germ, and endosperm |  |
| **Unfavorable to gut microbiota** |  |  |
| High-fat diet (% energy) | Not applicable | 0 if consumption at or above 40% energy from fat, else 1 For each remaining component, a score of 0 if consumption at or above the sex-specific median, else 1 |
| Processed meat | Frankfurters, sausages, corned beef, and luncheon meat that are made from beef, pork, or poultry |  |
| Red meat | Beef, veal, pork, lamb, and game meat; excludes organ meat and cured meat |  |
| Refined grains | Refined grains that do not contain all of the components of the entire grain kernel |  |
