# Supplementary material for: The association between dietary index for gut microbiota and colorectal cancer prevalence in US adults: Findings from NHANES 2003 to 2023
Source: Medicine (Baltimore). 2026 Jul 31;105(31):e49915. doi: 10.1097/MD.0000000000049915 (PMC13433049; doi:10.1097/MD.0000000000049915)
Supplement: Supplementary file 2 [file medi-105-e49915-s002.docx]

| **Table S2. Association between DI-GM and colorectal cancer after multiple imputation, NHANES 2003–2023** | | | | |
| --- | --- | --- | --- | --- |
| **Exposures** | **Colorectal cancer** | | | |
|  | **Model 1*** | | **Model 2**** | |
|  | **OR (95% CI)** | **P value** | **OR (95% CI)** | **P value** |
| **DI-GM (continuous)** | 0.88 (0.82, 0.94) | <0.001 | 0.93 (0.88, 0.99) | 0.024 |
| **Beneficial to gut microbiota** | 0.84 (0.74, 0.95) | 0.006 | 0.90 (0.78, 1.02) | 0.081 |
| **Unfavorable to gut microbiota** | 1.07 (0.91, 1.25) | 0.402 | 1.02 (0.86, 1.21) | 0.812 |
| **Abbreviations:** CI, confidence interval; DI-GM, dietary index for gut microbiota; NHANES, National Health and Nutrition Examination Survey; OR, odds ratio; CRC, colorectal cancer.  ***Model 1.** not adjusted. **Model 2. adjusted for age, sex, ethnicity, poverty income ratio (PIR), education level, body mass index, smoking status, physical activity, alcohol consumption, diabetes status, and total energy intake. Multiple imputation using chained equations was applied to handle missing data on covariates, assuming data were missing at random. | | | | |
